# Prediction of Obliteration After the Gamma Knife Radiosurgery of Arteriovenous Malformations Using Hand-Crafted Radiomics and Deep-Learning Methods

**DOI:** 10.7759/cureus.58835

**Published:** 2024-04-23

**Authors:** David J Wu, Megan Kollitz, Mitchell Ward, Rajiv S Dharnipragada, Ribhav Gupta, Luke T Sabal, Ayush Singla, Ramachandra Tummala, Kathryn Dusenbery, Yoichi Watanabe

**Affiliations:** 1 Medicine, University of Minnesota School of Medicine, Minneapolis, USA; 2 Radiology, University of Minnesota School of Medicine, Minneapolis, USA; 3 Neurosurgery, University of Minnesota School of Medicine, Minneapolis, USA; 4 Neurological Surgery, University of Minnesota School of Medicine, Minneapolis, USA; 5 Computer Science, Stanford University, Stanford, USA; 6 Radiation Oncology, University of Minnesota, Minneapolis, USA

**Keywords:** predictive models, convolutional neural network, radiomics, avm, radiosurgery, gamma knife

## Abstract

Introduction: Brain arteriovenous malformations (bAVMs) are vascular abnormalities that can be treated with embolization or radiotherapy to prevent the risk of future rupture. In this study, we use hand-crafted radiomics and deep learning techniques to predict favorable vs. unfavorable outcomes following Gamma Knife radiosurgery (GKRS) of bAVMs and compare their prediction performances.

Methods: One hundred twenty-six patients seen at one academic medical center for GKRS obliteration of bAVMs over 15 years were retrospectively reviewed. Forty-two patients met the inclusion criteria. Favorable outcomes were defined as complete nidus obliteration demonstrated on cerebral angiogram and asymptomatic recovery. Unfavorable outcomes were defined as incomplete obliteration or complications relating to the AVM that developed after GKRS. Outcome predictions were made using a random forest model with hand-crafted radiomic features and a fine-tuned ResNet-34 convolutional neural network (CNN) model. The performance was evaluated by using a ten-fold cross-validation technique.

Results: The average accuracy and area-under-curve (AUC) values of the Random Forest Classifier (RFC) with radiomics features were 68.5 ±9.80% and 0.705 ±0.086, whereas those of the ResNet-34 model were 60.0 ±11.9% and 0.694 ±0.124. Four radiomics features used with RFC discriminated unfavorable response cases from favorable response cases with statistical significance. When cropped images were used with ResNet-34, the accuracy and AUC decreased to 59.3 ± 14.2% and 55.4 ±10.4%, respectively.

Conclusions: A hand-crafted radiomics model and a pre-trained CNN model can be fine-tuned on pre-treatment MRI scans to predict clinical outcomes of AVM patients undergoing GKRS with equivalent prediction performance. The outcome predictions are promising but require further external validation on more patients.

## Introduction

Brain arteriovenous malformations (bAVMs) are uncommon congenital vascular lesions with aberrant arteries connecting directly into a venous outlet, resulting in direct arteriovenous shunting [1]. Complications of bAVMs include intracranial hemorrhage, seizures, mass effect, and steal phenomena, with rupture and subsequent hemorrhage being the most common and severe consequence [1-3]. Though rare, rupture and subsequent hemorrhage can result in significant morbidity and mortality, with mortality rate after rupture around 10-15% and morbidity rate 30-50% [1,2]. Many bAVMs are treated to prevent rupture or re-rupture while minimizing the risk of treatment. One treatment approach is stereotactic radiosurgery (SRS), particularly for small to medium AVMs deep within the brain parenchyma, and SRS can benefit those who are poor candidates for microsurgical resection or as an adjunct therapy [1,4].

SRS is a relatively new treatment approach for AVM compared with other treatments like microsurgery and endovascular techniques [5-8]. Leksell Gamma Knife (Elekta, Stockholm, Sweden) is widely used for the SRS of bAVM [9] and can achieve around a 75% obliteration rate [10]. Despite the excellent prognosis of SRS for bAVM treatment, the success and occurrence of side effects are highly dependent on patients. Hence, predicting which patients will experience the best outcomes with SRS treatment of their bAVMs is necessary. Previous studies have shown that higher doses and the absence of pre-radiosurgical embolization were independent predictors of the obliteration rate, while smaller volumes of the nidus and lower Virginia Radiosurgery AVM Scale (VRAS) were potential predictors of long-term favorable outcomes [11]. Scoring systems such as the Spetzler-Martin (SM) scale have been developed to characterize the surgical risk associated with AVMs; however, there is little evidence for the usefulness of these scales in adequately representing the relative risks of SRS [12, 13]. For example, the area-under-curve (AUC) values with those scoring systems ranged between 0.57 and 0.69 in the Oermann study [12], suggesting the need for more reliable methods for clinical decision-making.

Our aim, therefore, was to develop methods for predicting favorable vs. unfavorable outcomes following SRS treatment of brain AVMs using pre-treatment patient MRI scans and machine-learning models. Radiological images can be used as biomarkers in addition to standard clinical prognostic factors to improve the outcome prediction performance for SRS of bAVM. Radiomics using hand-crafted features has been used to build outcome prediction models of bAVM SRS by several groups [12,14-16]. However, deep learning approaches have not been used previously as a prediction model for this disease type. Hence, we fine-tuned ResNet-34, a convolutional neural network (CNN) [17], to build a prediction model for bAVM treatment by GKRS and then used hand-crafted radiomics features with the Random Forest Classifier (RFC) to create a similar model. We compared the performance of the CNN and radiomics prediction models.

## Materials and methods

Patient data

One hundred twenty-six patients who were seen for Gamma Knife SRS obliteration of bAVMs from 2006 to 2021 at the University of Minnesota Medical Center were reviewed retrospectively. Demographic data, clinical information, embolization and SRS details, complications, and follow-up information were collected. The patient characteristics are summarized in Table 1. Research approval was obtained from the Institutional Review Board (IRB-0810M23942).

**Table 1:** Patients’ characteristics The average and standard deviation are listed with the range in parentheses when applicable. P-values were calculated with the T.TEST function of MS Excel with two-tails and two-sample unequal variance type.

Characteristic	Whole set	Favorable	Unfavorable	p-value
No. of patients	42	25	17	
Male	25	14	11	
Female	17	11	6	
Age (Year)	44.0 ± 14.9 (12-73)	45.5 ± 16.0 (12-73)	41.9 ± 5.99 (22-68)	0.433
Volume (cm^3^)	4.84 ± 5.99 (0.08-28.10)	3.53 ± 4.46 (0.08-16.86)	6.77 ± 7.34 (0.11-28.10)	0.116
Margin dose (Gy)	18.6 ± 2.7 (12-24)	19.3 ± 2.5 (12-24)	17.6 ± 2.7 (12-23)	0.0473
Prescription isodose (%)	49.9 ± 3.3 (40.0-65.0)	49.8 ± 4.2 (40.0-65.0)	50.0 ± 0.0	
No. of prior embolization	13 (31.0%)	6 (29.2%)	6 (41.2%)	
Nidus type				
Compact	42	25	17	
Diffuse	0	0	0	
Nidus location				
Eloquent area	25	13	12	
Non-eloquent area	14	10	4	
Not determined	3	2	1	
Spetzler-Martin grade				
Grade 1	5	4	1	
Grade 2	9	6	3	
Grade 3	10	7	3	
Grade 4	8	3	5	
Grade 5	1	0	1	
Grade unknown	9	5	4	
Follow-up (months)	41.9 ± 35.8 (3.7-152.6)	44.8 ± 38.6 (7.9-152.6)	37.5 ± 31.8 (3.7-128.1)	0.515

Outcome classification

Favorable outcomes were defined as complete AVM obliteration demonstrated on cerebral angiogram and good neurological function (Modified Rankin score, mRS <2) [18]. Unfavorable outcomes were defined as incomplete obliteration or complications, including death, hemorrhage, or neurological deficits related to the radiosurgery. Originally, 43 patients met the inclusion criteria, but one additional case was excluded because CT was used instead of MRI for treatment planning, resulting in 42 patients (25 favorable and 17 unfavorable responses).

Gamma knife radiosurgery

Gamma Knife Radiosurgery (GKRS) was performed using the Leksell Gamma Knife Model 4C (before 2019) and Icon (after 2019). We followed the standard hospital treatment protocol for all treatments. All patients had pre-treatment T1-weighted MRI with contrast after a neurosurgeon placed the Leksell G-frame. After the MRI, the patient was transported for angiography with the fiducial box to acquire anterior-posterior and lateral angiographic images. A radiation oncologist and a neurosurgeon segmented target volumes on the MR images with the help of the images of nidus projected from the anteroposterior and lateral angiographic images. Note that 16 patients (38.1% of 42) had angiographic procedures before GKRS. For those patients, the angiographic data was indirectly utilized for target segmentation. The treatment plan was generated so that the prescription isodose surface, ranging from 12 to 24 Gy, covered the nidus without margin.

ResNet-34

**Figure 1:**
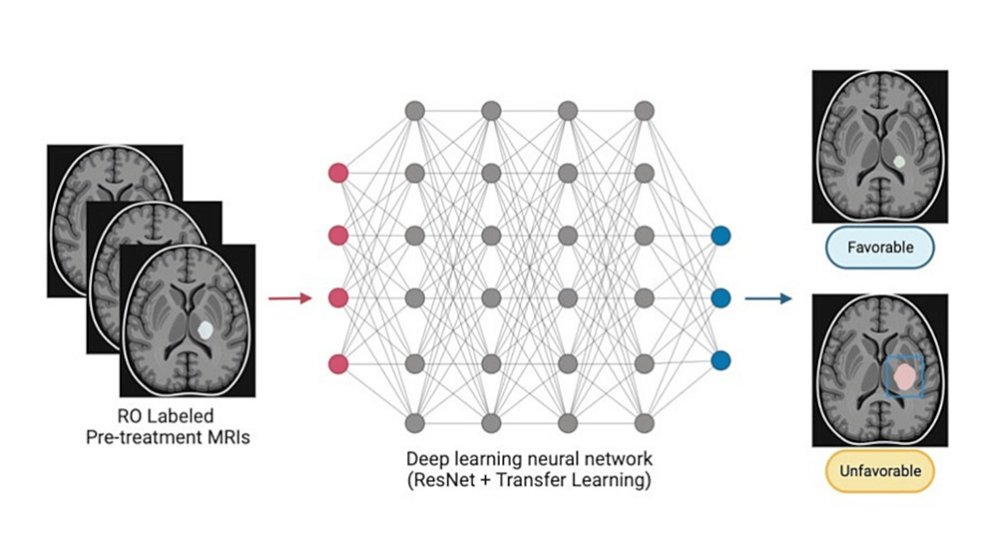
CNN-based deep learning pipeline CNN: convolutional neural network

For our deep learning approach, we adopted a ResNet-34 model, a type of CNN used ubiquitously in contemporary applications for medical image processing [19,20]. The ResNet-34 model is pre-trained on the ImageNet-21k benchmark [21], containing 14 million natural images spanning approximately 21,000 classes. We adopted the transfer learning paradigm for model training due to the minimal size of our dataset and fine-tuned this model on a collection of two-dimensional (2D) MRI slices (ResNet-34 is limited to 2D images). We manually selected a single MRI slice for each patient, choosing the slice with the largest AVM dimension among the slices containing an AVM contour. This model was implemented using PyTorch, a deep-learning library for Python [22]. Each image was then associated with a corresponding outcome, 0 for unfavorable and 1 for favorable. See Figure 1 for the deep learning-based modeling procedure.

For the second analysis, the patients were randomly split into training and testing datasets using an 80:20 split for training and evaluating the model. The training set contained 34 patients, and the test set contained eight patients. The model was trained for 34 patients over 20 epochs. Since splitting data into training and testing sets is random, and the test set includes only a small number of patients, the test results are susceptible to selection bias in which patients were chosen with the ratio of unfavorable and favorable patients in the test set. Hence, to remediate this issue for the second analysis, we evaluated the model performance using the shuffle split cross-validation, also known as the repeated random sub-sampling validation or Monte Carlo cross-validation [23]. We randomly divided the data of 42 patients into training and test sets with 67% and 33 % or 29 and 13 patients for training and testing, respectively, and then repeated this procedure 10 times. The final performance results were evaluated by calculating the averages of the accuracy of the prediction and the AUC value of the receiver-operating characteristic (ROC) curves.

ResNet-34 automatically focused on the potential disease area, and it did not use the segmentation data of the AVM. Hence, the prediction performance likely depends on the region-of-interest (ROI) size used for the training and testing. To study this effect, we generated cropped images that included the AVM contour at the center with a limited size of 60 x 60 mm^2^. We used these cropped images to build a CNN model and evaluate the prediction performance using the shuffle split cross-validation, repeating 10 times.

Radiomics

A predictive model can also be built using radiomics features as predictors. We calculated 1038 radiomics features of 42 patients using SlicerRadiomics, PyRadiomics version 3.0.1 [24], implemented in the 3D Slicer software [25]. The feature calculation grid size was set to 1 mm x 1 mm x 1 mm. There were 107 original radiomics features in shape (14), first order (18), and texture (75) groups. The texture group included 24 features of the Gray-level-co-occurrence matrix (glcm), 14 the Gray-level domain matrix (gldm), 16 Gray-level run length matrix (glrlm), 16 Gray-level size zone matrix (glszm), and 5 Neighborhood gray-tone difference matrix (ngtdm). After applying the Laplacian-of-Gaussian filter with kernel sizes of 1 mm and 3 mm and wavelet filters, the total number of features increased to 1038.

**Figure 2:**
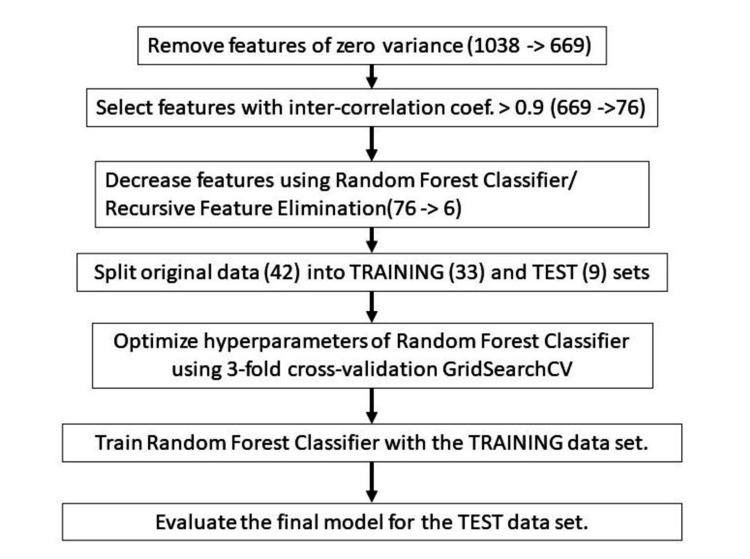
Predictive modeling and evaluation procedures with radiomics This image was drawn by the authors of this article.

The radiomics-based model generation and evaluation procedures are illustrated in Figure 2. We applied a three-step feature reduction method to reduce the number of radiomics features to prevent overfitting of the models. First, the features with a standard deviation smaller than 0.3 were removed. Intercorrelation coefficients among the remaining 669 features were calculated. The features with correlation coefficients greater than 0.9 were eliminated to decrease the total number to 76. We used the recursive feature elimination method with the RFC to further reduce the number of features. This process reduced the number to six.

The random forest model contains several model-specific hyperparameters, such as maximum depth, maximum features, minimum sample leaf, minimum sample splits, and the number of estimators. An optimized set of hyperparameters was obtained using the grid search technique with the training data set after randomly splitting the 42 patients into two groups: 80% of the data (33 patients) for training and 20% for testing (9 patients).

The model’s performance was evaluated in three ways. For the first method, we used the training data and applied the stratified 10-fold cross-validation technique using 80%/20% of patient data for training and validation. The stratified method was used to minimize the negative effects of unbalanced numbers of unfavorable and favorable response patients in the training and validation data sets. Secondly, the prediction model’s performance with the optimized hyperparameter values was evaluated with the test data set of nine patients. Lastly, we used the shuffle split cross-validation method by randomly dividing 42 patients into training and test sets with 67% and 33% ratios (or 28/14 patients) in training and test sets, respectively. This evaluation process was repeated 10 times to obtain the average prediction performance of the model. Additionally, to find the most relevant radiomics features that indicated strong relationships with the outcome among all the features, we ranked the features by calculating the performance of the RFC for randomly selected features with increasing numbers of features.

Modelling and statistical analysis

All computational tools to construct predictive models were taken from Python packages such as sklearn (scikit-learn) and PyTorch. Radiomics analysis was done with 3DSlicer. The model was evaluated using the ROC curves with AUC values and calculating the confusion matrix and the accuracy. Most of the analysis in this study was done using Python libraries. The student t-test was done with the function available on the Microsoft Excel program.

## Results

Patient, AVM, and treatment characteristics

Table 1 summarizes the characteristics of patients (gender, age, follow-up), AVM (volume, nidus type, nidus location, prior embolization, SM grades), and dosimetry (margin dose, prescription isodose line) for all 42 patients. Among them, 25 patients had favorable responses, and 17 had unfavorable responses, indicating a 59.5% success rate. P-values comparing two response types are shown when appropriate. Note that the follow-up time was between the end of GKRS and the last clinical visit, which was used to decide the response type. The average follow-up time was 41.9 months. Forty-one patients had greater than three years of follow-up. All nidi were compact type. 58% of those were in an eloquent area. Six patients with favorable responses (29.2%) and seven with unfavorable responses (41.2%) had embolization before GKRS. The SM grades varied from grades 1 to 5. Most AVMs were grade 3 and 4 for both favorable and unfavorable cases. There was no difference in age and volume between the two groups.

As for dosimetry, the AVM of the unfavorable group had a smaller margin dose than the favorable group with p = 0.047. There was no difference in the prescription isodose line. The difference in the prescription dose stems from the difference in the target volume. We prescribed a lower dose for a larger volume; the volumes with unfavorable responses had larger volumes.

ResNet-34

**Figure 3:**
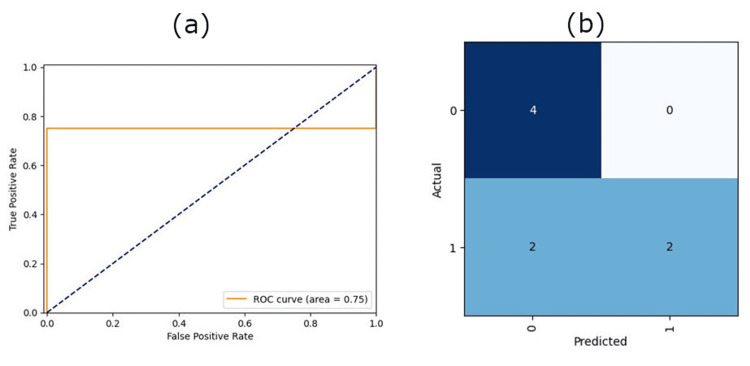
ResNet-34 results for eight test patients (a) ROC, (b) Confusion matrix ROC: receiver-operating characteristic This image was drawn by the authors of this article.

Final validation of the ResNet-34 model on eight patients showed an accurate prediction on six patients with a final accuracy of 75% and an AUC of 0.75. See Figure 3 for ROC and confusion matrix. The model’s sensitivity was 50.0%, meaning that the prediction model correctly labeled a favorable outcome as favorable half of the time. The specificity was 100.0%, meaning the model marked an unfavorable outcome unfavorable 100% of the time. Given the risk/benefit ratio of incorrectly labeling a favorable outcome as an unfavorable outcome versus incorrectly labeling an unfavorable outcome as a favorable outcome, maximizing sensitivity over specificity may be prioritized.

The shuffle split cross-validation resulted in an average accuracy of 60.0 ± 11.9% and an average AUC of 0.694 ±0.124. When cropped images were used for the modeling, the performance changed to an accuracy of 59.3 ± 14.2% and AUC of 0.554 ±0.104. The p-value of AUC was 0.015, indicating that the decrease in the AUC with cropped images was significant. Please refer to Table 2 for more details on the model’s performance for each fold.

**Table 2:** Prediction performance of Random Forest classifier (RFC) with radiomics and ResNet-34 models for repeated training and testing with full and cropped MR images The accuracy and AUC of RFC and ResNet-34 did not differ statistically for full images (p=0.098 and 0.830 for accuracy and AUC, respectively.) Moreover, there was no difference in accuracy between ResNet32 results with full and cropped images (p=0.903), but AUC was statistically different with p=0.015. The p-values were calculated using the T.TEST function of MS Excel with a two-tailed tail and two-sample unequal variance type.

Fold	RFC		ResNet-34 (full image)		ResNet-34 (cropped image)	
	Accuracy	AUC	Accuracy	AUC	Accuracy	AUC
1	0.790	0.822	0.462	0.567	0.550	0.500
2	0.710	0.733	0.538	0.700	0.750	0.690
3	0.640	0.735	0.769	0.833	0.750	0.670
4	0.710	0.733	0.615	0.700	0.625	0.500
5	0.710	0.667	0.615	0.786	0.375	0.500
6	0.790	0.750	0.538	0.571	0.500	0.570
7	0.790	0.733	0.692	0.567	0.375	0.330
8	0.570	0.521	0.692	0.700	0.625	0.670
9	0.500	0.604	0.692	0.925	0.625	0.580
10	0.640	0.750	0.385	0.595	0.750	0.533
Average	0.685	0.705	0.600	0.694	0.593	0.554
Std dev	0.0980	0.0860	0.1190	0.1240	0.1424	0.1084

Radiomics model

**Figure 4:**
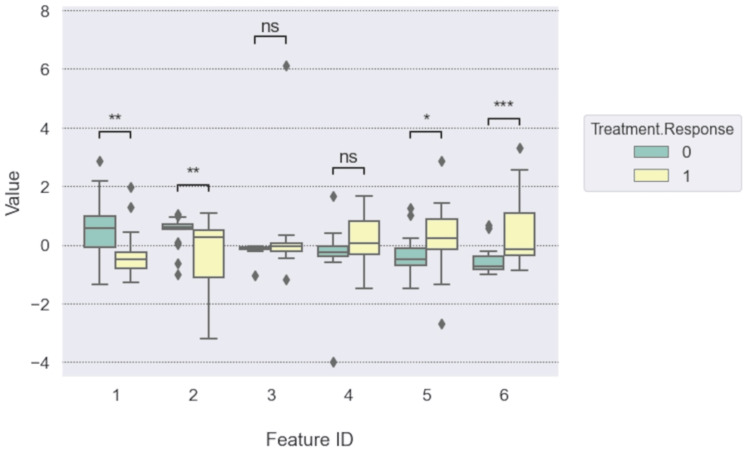
Differences of radiomics feature values between unfavorable (“0”) and favorable (“1”) responders The numbers of the x-axis label correspond to six radiomics features selected for the final model. 1: “original gldm Large Dependence High Gray-Level Emphasis”, 2: “wavelet-LLH glcm Cluster Shade”, 3: “wavelet-LHH first order Mean”, 4: “wavelet-HLH first order Mean”, 5: “wavelet-LLL first order Skewness”, and 6: “wavelet-LLL ngtdm Strength”. The statistical significance levels are indicated by “ns”: p > 0.05, “*”: 0.01 < p < 0.05, “**”: 0.001 < p < 0.01, and “***”: 0.0001 < p < 0.001. This image was drawn by the authors of this article.

Table 3 shows the top 15 ranked features, including the six features (rank no. 1) that were used for the prediction model as the top-ranked features. There are five non-wavelet-based features among these 15 features. The box-Whisker plot in Figure 4 shows that the differences of four radiomics features among six selected for the predictive model (1: “original gldm Large Dependence High Gray-Level Emphasis”, 2: “wavelet-LLH glcm Cluster Shade”, 5: “wavelet-LLL first order Skewness”, and 6: “wavelet-LLL ngtdm Strength”) were statistically significant (p<0.05) between unfavorable “0” and favorable “1” responders. The results imply that the images of unfavorable responders were more nonuniform, whereas favorable responders had primitives (or small areas) distinguishable from the rest of the imaged area.

**Table 3:** Ranking of radiomics features

Feature name	Rank
wavelet-LLL ngtdm Strength	1
wavelet-LHH firstorder Mean	1
wavelet-LLH glcm ClusterShade	1
original gldm LargeDependenceHighGrayLevelEmph...	1
wavelet-LLL firstorder Skewness	1
wavelet-HLH firstorder Mean	1
original firstorder Skewness	2
wavelet-LLL ngtdm Busyness	3
log-sigma-1-0-mm-3D glcm ClusterShade	4
wavelet-LLH firstorder Mean	5
original glcm ClusterShade	6
wavelet-HHH firstorder Mean	7
wavelet-LLL glszm LargeAreaHighGrayLevelEmphasis	8
wavelet-LHL glcm ClusterShade	9
wavelet-LLL firstorder Kurtosis	10

**Figure 5:**
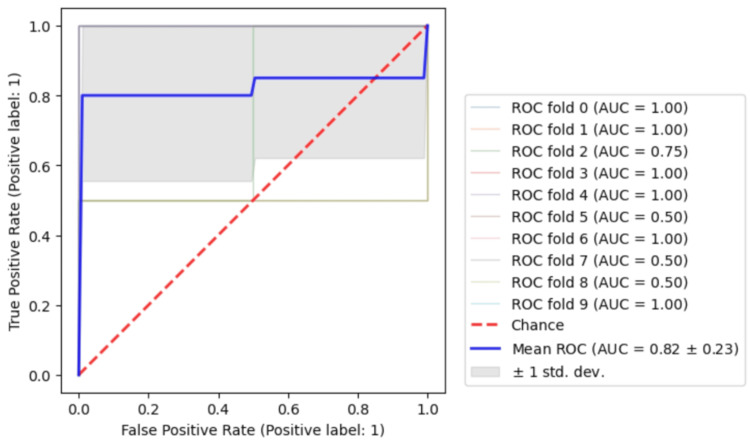
ROC of 10-fold cross-validation using Random Forest Classifier with 33 patients training data ROC: receiver-operating characteristic This image was drawn by the authors of this article.

**Figure 6:**
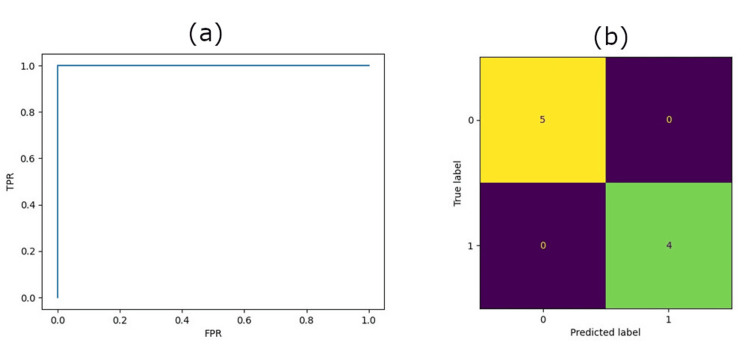
Radiomics results for nine test patients (a) ROC, (b) confusion matrix ROC: receiver-operating characteristic This image was drawn by the authors of this article.

The stratified 10-fold cross-validation showed that the RFC models could achieve an average AUC of 0.82±0.23 as shown in Figure 5. The accuracy and AUC values of the model were 97.0% and 0.992 for the training set (33 patients, 12 unfavorable, 21 favorable). In contrast, those were 100% and 1.0, respectively, for the test data set (9 patients, five unfavorable, and four favorable). See Figure 6 for ROC and confusion matrix. The shuffle split cross-validation resulted in an average accuracy and AUC value of 68.5 ± 9.80% and 0.705 ± 0.086, respectively. These did not differ statistically from the ResNet-34 results with full MR images (Table 2).

## Discussion

This study is the first attempt to apply a sophisticated machine-learning model to predict the outcomes of AVM after radiosurgery. The model used was the ResNet-34, a type of CNN. Since we had a minimal number of cases for training (n < 42), we adopted a transfer learning approach in which the model was pre-trained on the ImageNet-21k benchmark. The transfer learned ResNet-34 performed reasonably well with our data. Its performance was comparable with the RFC using radiomics. Both models performed equally to predict the response of bAVM to GKRS.

An advantage of the radiomics-based method over the CNN method is its potential ability to provide disease-specific characteristics of images, leading to valuable insights for understanding biologically based outcome predictions. The current study indicated that many radiomics features calculated using wavelet-filtered MR images were promising biomarkers for the prediction.

Other groups have also published their work applying machine-learning-based models for outcome predictions. Oermann et al. comprehensively analyzed 1674 patients with GKRS for cerebral AVM but did not use radiomics techniques; instead, they constructed prediction models using traditional methods such as logistic regression, linear support vector machine, random forest, and also built models using standard prognostic factors such as the SM grading scale, the modified radiosurgery-based AVM score (BRAS), and the Virginia Radiosurgery AVM Scale (VRAS) [12]. They found that their selected predictive features resulted in a better performance than the standard parameters of SM, BRAS, and VRAS, demonstrating the importance of well-selected hand-crafted features for outcome predictions of AVM after radiosurgery. Recently, Gao et al. used radiomics for outcome predictions of AVM treated by GKRS using the data of 88 patients [16]. Their study used only the random forest model and did not use deep learning tools and achieved an impressive AUC of 0.88 using 12 selected radiomics features. Furthermore, they demonstrated the predictive model using typical clinical quantities (age, gender, size of AVM, nidus location, etc.), and the SM-BRAS scores performed poorly in line with the Oermann et al. results.

Comparing our results with other studies, we achieved a post-treatment AVM control rate of 59.5%. This result aligns with other studies, such as the 63.6% success rate of the Gao et al. study [16], which did linear accelerator-based AVM radiosurgery, as well as the 58% obliteration rate for medium volume sizes, 4-13.9 cm^3^ found in Miyawaki et al.’s study [6]. However, some groups had higher success rates, indicating obliteration rates as high as 79% [26]. Hence, our data shows an average treatment success rate of AVM, with room for improvement.

There are several limitations in the current study. First, the number of cases was small. Though a transfer learning paradigm helps mitigate this issue, the current model’s performance could likely be improved significantly by increasing the training set. This can be done via techniques such as data augmentation which have been shown to be both effective and less-labor intensive than generating new data [27]. Second, the CNN-based model used a 2D slice of MRI with the AVM segmentation due to this study’s limitation of the ResNet-34 routine. The predictive ability may be significantly improved using the entire three-dimensional (3D) image data with the model training [28]. Third, the current CNN model was built using a whole 2D slice image with the malignant area occupying a small portion, so the algorithm needed to find the AVM automatically while the contour data of the malignant area was not used for the analysis. To help the software recognize the AVM, we tested smaller sizes of cropped images with sufficient margins around the tumor for model building. The performance did not improve, but rather, it degraded. Fourth, a well-known disadvantage of any CNN model is its lack of easily recognizable predictors. Some investigators, however, have successfully extracted these predictors, called deep features, from trained deep-learning models [29]. Lastly, as for the radiomics-based model, potentially valuable features for outcome prediction were found, indicating differences in the image non-uniformity and texture complexity between the two responders. Their biological meaning must be yet to be clarified to make those predictions more meaningful for clinical applications.

## Conclusions

Pre-trained CNN models can be fine-tuned on pre-treatment MRI scans to predict clinical outcomes of AVM patients undergoing SRS ablation. The deep learning-based model achieved predictive performance comparable to the hand-crafted radiomics model. The outcome predictions are promising but require further validation on more patients and data from external institutions to assess these predictions’ accuracy and reliability. Future directions would ideally involve multicentric, prospective studies to verify the models' applicability across diverse patient populations and imaging modalities. The models themselves could likely be further enhanced by using 3D imaging data to provide a more nuanced understanding of AVM characteristics and treatment responses.
